# Research in the Commonwealth of Independent States on Superconducting Materials: Current State and Prospects

**DOI:** 10.3390/ma18184299

**Published:** 2025-09-13

**Authors:** Sanat Tolendiuly, Adil Akishev, Sergey Fomenko, Jaafar Nur-Akasyah, Abu Bakar Putra Ilhamsyah, Nursultan Rakhym

**Affiliations:** 1 Institute of Combustion Problems, Almaty 480012, Kazakhstan; s.tolendiuly@aues.kz (S.T.); adilak1943@gmail.com (A.A.); exotherm@yandex.kz (S.F.); 2 Department of Chemistry, Kulliyyah of Science, International Islamic University Malaysia, Kuantan 25200, Pahang, Malaysia; jnurakasyah@gmail.com; 3 Department of Applied Physics, Universiti Kebangsaan Malaysia, Bangi 43600, Selangor, Malaysia; ilhamsyahputraabubakar@gmail.com; 4 Department of Solid State Physics and New Materials Technology, Al-Farabi Kazakh National University, Almaty 050040, Kazakhstan

**Keywords:** HTS, LTS, Commonwealth of Independent States, manufacturing, superconducting properties

## Abstract

An overview of research on superconducting materials has been provided, including brief annotations of published papers and scientific cooperation among the Commonwealth of Independent States (CIS) countries: Armenia, Azerbaijan, Belarus, Kazakhstan, Kyrgyzstan, Moldova, Russia, Tajikistan, Turkmenistan, Ukraine, and Uzbekistan. It is shown that fundamental research on superconducting materials is being funded for development and study more at the government level in each republic than from private funds or organizations. One of the most promising materials, as indicated by recent studies, are those synthesized from metal hydrides, particularly lanthanum hydride, which exhibits superconducting properties at 203–253 K, close to room temperature. Unfortunately, this type of material’s practical application is currently limited because of the extremely high pressure necessary during exploitation. The most promising direction, as inferred from research conducted in CIS countries, is the development of cuprate superconductors doped with rare-earth elements such as yttrium, lanthanum, and other metals. There are also iron–nitrogen junctions, metallic and organic superconductors, and research into improving technologies for producing ultrathin substrates using laser or plasma deposition methods. CIS countries have established a strong scientific foundation in superconductivity, with Russia leading fundamental and experimental advances in high- and low-temperature superconducting materials. Future research will likely focus on improving synthesis techniques for ultrathin superconducting films and exploring novel doped hydride systems to achieve stable superconductivity near ambient temperatures.

## 1. Introduction

Technological progress is closely linked to the development of new materials that can endure the specific conditions of their application. One of the key areas in materials science, as well as in solid-state and theoretical physics, is the investigation of crystal structures, lattice parameters, and the physical processes that occur in materials at ultra-low temperatures, particularly those that exhibit superconducting properties.

Comprehensive studies of superconducting materials, starting from the time of their discovery and up to the present time, using substances and compounds with different physical properties and electronic structures, have shown that, depending on the critical temperature and cryogenic environment, they can be divided into low- [1], high- [2] and medium-temperature superconductors (materials with critical temperatures between LTS and HTS ranges).

Low-temperature superconductors are substances with a transition to a superconducting state at a temperature of <23.2 K. These include niobium-titanium alloy and intermetallic-based superconductors such as Nb_3_Sn, V_3_Ga, Nb_3_Al, etc. [3].

As for high-temperature superconductors (HTS), they can be divided into the first and second generations. Examples of HTS materials: BSSCO (critical temperature 125 K, commonly related to 1G), YBCO (*T_c_* about 93 K, commonly related to 2G), mercury (*T_c_* at 133 K) and thallium (*T_c_* of around 125 K) based superconductors. The first generation of HTS with a critical temperature of 110 K includes multi-fiber tape composites [4]. The second generation of HTS are multilayer tapes made of a metal carrier with buffer layers with a critical temperature of 93–95 K [5] and currently the most common HTS is made in foam format, with uses, for instance, in the aerospace industry [6].

Meanwhile, medium-temperature superconductors with a transition temperature between 20 and 53 K include pnictides (iron-containing chemical compounds with *T_c_* up to 53 K) and compounds based on magnesium diboride (MgB_2_ with *T_c_* at 39 K) [7]. The critical temperature and the critical magnetic field parameters of MgB_2_ usually remain constant due to the crystallographic nature of these superconductors, regardless of synthesis methods or other types of chemical modification of microstructure. *T_c_* values in iron pnictides are sensitive to chemical modification. However, the critical current density is associated with the structure of the superconducting phase and can be modified using technological methods.

Research on superconducting materials has been conducted in the Commonwealth of Independent States (CIS), which has led to rapid development in the electronic fields, the nuclear industry, and new energy sources. CIS is an alliance of eleven member states, currently, which emerged in 1991 from the former republics of the Soviet Union and are all located in Asia and Eastern Europe. At present, the CIS unites: Armenia, Azerbaijan, Belarus, Kazakhstan, Kyrgyzstan, Moldova, Russia, Tajikistan, Turkmenistan, Ukraine, Uzbekistan. This progress has also provided additional impetus for advancements in thin-film technologies, theoretical physics, plasma physics, and laser technology.

## 2. Research Finding

Superconductivity is a unique quantum phenomenon characterized by the complete absence of electrical resistance in certain materials when subjected to ultra-low critical temperatures (*T_c_*) under these conditions, energy losses are reduced to mere fractions of a percent [1].

Since the discovery of superconductivity, research in this field has been conducted worldwide, with significant contributions from Soviet scientists across various republics. Notably, after the dissolution of the Soviet Union and the formation of the CIS, research on superconductors and their improvement has continued, as evidenced by numerous publications and ongoing collaborations among the member states [4].

The study of superconductivity has shown that they have unique diamagnetic properties—the ability to push a magnetic field out of its volume (the Meissner effect) [8] including the effect of levitation (which is one of the criteria for confirming superconductivity). Nowadays, the most popular way of identifying superconducting parameters of *T_c_* is a resistance measurement as a function of temperature.

The discovery of HTS with the advent of ceramic materials, such as lanthanum-barium-copper oxide (La_2−_*_x_*Ba*_x_*CuO_4_) [9,10] and yttrium-barium-copper oxide (YBa_2_Cu_3_O_7−*x*_) [11,12,13], provided a way out of some stagnation in superconductor research.

The CIS countries possess significant potential for the development and application of new types of superconductors, which play a crucial role in advancing the physicochemical properties of materials and their use in various industries and applications.

The purpose of this work is to review existing studies on superconducting materials conducted in the CIS. This work also aims to study currently promising directions of developed compositions, structures, and properties of new superconducting materials. And lastly, to acknowledge established technological features contributed by the superconducting materials, as well as the theoretical principles behind them.

## 3. Superconductivity Research in the Commonwealth of Independent States (CIS)

Most of the leading research centers in superconductors in the CIS countries are currently located in Russia, where they specialize in the synthesis of superconducting materials from LTS up to HTS with various directions of application. For instance, the Institute of Solid State Physics of the Russian Academy of Sciences (ISSP RAS) and the National Research Center “Kurchatov Institute” are actively involved in developing superconducting wires and films based on both low-temperature superconductors like NbTi and Nb_3_Sn, and high-temperature superconductors such as YBCO and BSCCO for applications in power grids, MRI systems, and particle accelerators [14].

In Ukraine, the main direction was the development of new compositions for HTS and improving the procedures for enhancing superconducting properties using modern devices. For instance, researchers at the Institute for Low Temperature Physics and Engineering of the National Academy of Sciences of Ukraine have conducted microscopic studies on YBa_2_Cu_3_O_7−*x*_ (YBCO) crystals. Their work revealed that the superconducting transition temperature varies across different microscopic regions of the crystal, influenced by structural inhomogeneities. This insight is crucial for optimizing fabrication techniques to enhance the superconducting properties of HTS materials [15].

Meanwhile, research in Kazakhstan is focused on obtaining superconductors based on cuprates and rare-earth elements to be used in mechanical engineering and space equipment applications. For example, active development is underway on the technological foundations for synthesizing electro-conductive cables using the solid-phase combustion method. These cables are intended for use in aerospace engineering, particularly as lightweight and efficient replacements for conventional wiring in spacecraft and aviation systems [16].

In the Republic of Belarus, work is actively underway to develop superconducting materials for the application of medical equipment like magnetic resonance imaging (MRI). For example, the Research Institute for Nuclear Problems of the Belarusian State University (INP BSU) focuses on materials science and nanotechnology, which are essential for advancing superconducting technologies used in MRI systems [17].

**Figure 1:**
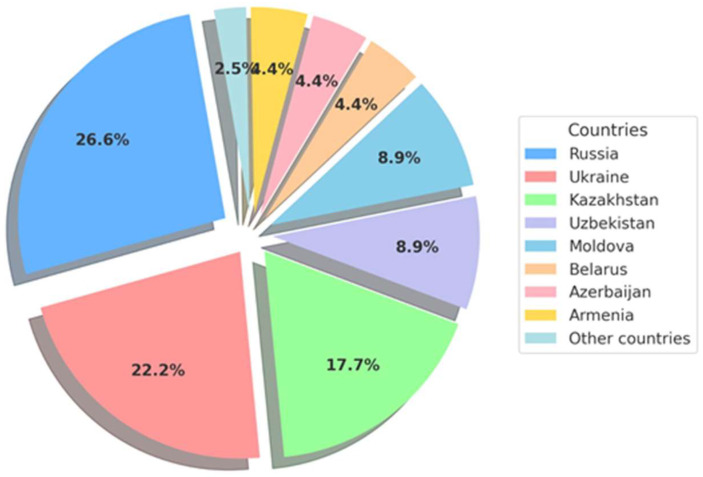
The distribution of research in the field of superconductors in the CIS.

In the CIS countries such as Armenia, Azerbaijan, Uzbekistan, Moldova, Tajikistan, Kyrgyzstan, Turkmenistan, research in the field of superconducting materials is less developed, but there are a number of promising projects that can be implemented in the near future (Table 1, Figure 1).

Research on superconductors should be especially noted, as they are mainly concentrated in the Russian Federation, which has significant achievements since the middle of the last century.

Table 1 provides a structured summary of superconductivity studies conducted in different CIS countries, indicating differences and synergies in institutional participation, research focus, materials studied, and key results. Each country demonstrates a unique focus in the broader field of superconductivity, ranging from fundamental physics (e.g., BCS theory, vortex dynamics) to practical applications (e.g., MRI technology, superconducting wires). Taken together, the data show that superconductivity research remains a dynamic field in the post-Soviet scientific landscape, with an active contribution to global scientific progress.

The pie chart in Figure 1 illustrates the distribution of research results in the field of superconductors across the countries of the Commonwealth of Independent States (CIS). These data reflect the percentage contribution of each country to the field of superconductivity, highlighting the relative levels of academic or scientific activity related to this field of physics. Superconductivity research is crucial for technological advances in areas such as quantum computing, energy transfer, and magnetic levitation systems. Understanding the distribution of research activities can help identify regional leaders and potential areas for increased collaboration or investment. The distribution of research in the field of superconductors in the CIS is mainly concentrated in Russia, Ukraine, and Kazakhstan, which together account for more than 66.5% of the total research volume. This reflects both historical and current investments in research infrastructure. Middle-level countries such as Uzbekistan and Moldova are showing promising results, while smaller contributions from other Member States suggest opportunities for growth through international cooperation, capacity building, and targeted financing. Overall, the data highlight regional differences, but also the collective potential of the CIS in advancing superconductivity research.

## 4. Notable Advances in Enhancing Superconducting Properties

Particular attention should be given to the works of the Russian scientist, Academician V. L. Ginzburg, under whose leadership superconductivity and superfluidity were studied for an extended period at the P. N. Lebedev Physical Institute (LPI) of the Russian Academy of Sciences. The superconducting materials studied at the P. N. Lebedev Physical Institute included various compounds of metals, alloys and ceramic materials, including Nb_3_Sn, Nb_3_Ge, YBa_2_Cu_3_O_7−*x*_, MgB_2_, (TMTSF)_2_PF_6,_ and others [21,37]. It was obvious that superconducting properties of HTS strongly depend on the composition, structure and chemical doping, as well as heating temperature, environment pressure during the synthesis process and applied magnetic field. The obtained materials exhibit high values of critical temperature (*T_c_*), critical density (*J_c_*) and critical magnetic induction (*B_c_*_2_). An unusual phase diagram was compiled and the multi-frequency Debye effect, asymmetric Cooper pairing, strong anisotropy and other studies were established [38]. These studies made it possible to use them in generators and detectors of terahertz radiation, in quantum computers and logic elements, solenoids and the inductor coil, as well as in magnetic systems for accelerators, in nuclear physics, sensors for astrophysics and medicine [39].

A. A. Abrikosov, V. Ginzburg and Anthony Leggett made a decisive contribution to the explanation of two phenomena of quantum physics: superconductivity and superfluidity [40].

Studying the magnetic field and its effect on superconducting materials [61], Schmidt D. B. in his work indicates that in most cases the magnetic field leads to the destruction of superconductivity. With a gradual increase in the magnetic field, the Meissner effect is first observed in superconductors, which consists of the fact that the external magnetic field penetrates the sample to a small distance. Then, when the critical value of the field is provided, the superconductor passes into a state with the simultaneous presence of normal and superconducting zones, resulting in a non-zero electrical resistance that is a combination of the normal state resistance in the normal zones and the resistance associated with the movement of vortices in the mixed state.

Continuing research in this field and based on the obtained results, high-temperature superconducting cuprates with reduced heavy metal content (Ba, Tl) were developed at the Ioffe Physical-Technical Institute of the Russian Academy of Sciences (St. Petersburg, Russia). It was established that in YBaCu_2_O*_7_*, the phase transition begins at 93 K and completes at 88 K, while in Tl_1.5_Ba_2_Ca_2_Cu_2.5_O_8_, the phase transition starts at 125 K and finishes at 90 K. The authors also demonstrated the formation of the Meissner effect, which is a characteristic property of superconductors. When immersed in liquid nitrogen, the samples and the magnet exhibited mutual repulsion at distances of 1.0 cm and 1.5 cm, respectively [41].

Recently, significant interest has been shown in hydrides, particularly in the developments by Troian I. A. (National Research Nuclear University) [42]. It is reported that a series of triple hydrides La-Y were synthesized under pressures of 170–196 GPa by laser heating of La-YH_6_ alloys. The cubic hexahydride (La,Y)H_6_ and decahydrides (La,Y)H_10_ were discovered, exhibiting a maximum critical temperature *T_c_* ≈ 253 K and an extrapolated upper critical magnetic field *B_c_*_2_(0) of up to 135 T at 183 GPa. These compounds also demonstrated a current density ranging from 12 to 27.7 kA/mm^2^ at 4.2 K, which is comparable to the current density of conventional superconducting wires such as NbTa and NbSn [42]. The compounds (La,Y)H_6_ and (La,Y)H_10_ represent some of the first known examples of ternary superconducting hydrides with high *T_c_*.

Another example is FeN_4_H_4_, which has a *T_c_* of ~10 K. Its structure consists of layers of iron and nitrogen separated by layers of hydride. When the temperature is lowered, the electrons in this compound also form Cooper pairs, but with higher binding energy. This allows a higher critical temperature to be achieved than in FeN. They can be used in new devices and applications, such as magnetic levitators, energy-saving transmission lines, and quantum computers [62].

The authors [27] found that in multilayer cuprate compounds, interlayer dielectric order couples the superconducting condensates of adjacent planes. This order significantly increases the superconducting transition temperature *T_n_* with the growing number *n* of copper-oxygen planes. The decrease in *T_n_* for *n* > 3 is associated with the breakdown of interlayer nesting and the suppression of interlayer dielectric order due to the mismatch of Fermi surface contours in adjacent planes with different doping levels.

Since the discovery of cuprate HTS, a vast number of neutronographic studies have been conducted, providing extensive information about their crystallographic and physical properties [12]. Neutronographic research has been primarily carried out at the Kurchatov Institute National Research Center, the St. Petersburg Institute of Nuclear Physics of the Russian Academy of Sciences (RAS), the Institute of Metal Physics of RAS, and the Joint Institute for Nuclear Research (JINR). Neutron scattering experiments on HTS have established that as the carrier concentration increases, the antiferromagnetic (AFM) ordering of copper spins in the undoped state rapidly collapses, and at a critical doping level *X_c_*, the Néel temperature drops to zero. When the doping level exceeds *X_c_*, incommensurate magnetism emerges in the superconducting phase. In the underdoped region, at temperatures higher than both the superconducting transition temperature *T_c_* and the Néel temperature T_n_, a pseudogap appears. This pseudogap is characterized by the opening of a gap in the electronic excitation spectrum.

Perovskite structural compounds [28] such as SrTiO_3_, BaTiO_3_, PbTiO_3_, and SrTiO_3_ (001) (111) have been obtained using hybrid descriptions and correlations (*B*_3_*PW* or *B*_3_*LYP*). Initial calculations in the perovskite structure show that atoms in the first upper layer tend to move into the material’s bulk, while atoms in the second layer move (relax) toward the surface. Surface energies in the (111) plane are significantly higher than those in the (001) plane. It was established that the covalent chemical bond in the bulk of these perovskites increases at the surface with the ends of BO_2_(001) and AO_3_(111).

One of the most exotic families of iron-containing superconductors with the *A*FeAs structural type (*A* = Li, Na) is reviewed in article [29]. The physical and electronic properties of LiFeAs and NaFeAs are briefly discussed, where their multi-gap superconducting state is established based on theoretical models of these pnictides. Specifically, it is shown that large high-quality single crystals of *A*FeAs (up to 1 cm in size) can be grown using the “melt solution” method [30,31]. However, for instance, when LiFeAs is exposed to open air, its critical temperature drops to zero within 10–20 min, with LiOH being released between crystalline blocks, and its surface begins to degrade with the appearance of oxygen and water vapor. Atmospheric nitrogen also starts to chemically react with *A*FeAs. Research has shown that a “dry” vacuum or an appropriate protective atmosphere is required during experiments to prevent the impact of the reactive environment.

In [32] this paper, the behavior of electrical resistance near the superconducting transition in the Du_0.6_Y_0.4_Rh_3.85_Ru_0.15_B_4_ compound is investigated for different orientations (*φ* = 0°, 45° and 90°) of *R*(*T*) in different external magnetic fields relative to the direction of the measuring electric current. The measurements of the electrical resistance R(T) of a textured polycrystalline Du_0.6_Y_0.4_Rh_3.85_Ru_0.15_B_4_ sample were performed in the temperature range of 2 ÷ 12 *K* and in magnetic fields up to 36 kOe. For angles *φ* = 0° and 45°, the experiments were carried out in magnetic fields *H* = 0–9 kOe, and for *φ* = 90°—at *H* = 0–36 kOe. The shape of the dependence *R*(*T*) in the field range of 0–6 kOe is typical for a superconducting transition: a sharp drop in resistance below a certain temperature with its subsequent disappearance at lower temperatures. A different type of behavior of *R*(*T*) was observed at *φ* = 45° in fields above 6 kOe. In this case, the resistance decreases only to a certain final value *R_mi_*_n_ ≈ 0.4 *R_N_* (*R_N_* is the resistance of the sample in the normal state), and then, with a further decrease in temperature, *R*(*T*) rapidly increases. With a further increase in the field, the observed minimum of *R*(*T*) shifts to the low-temperature region and decreases in depth to *R_min_* ≈ 0.9 *R_N_*. Thus, it can be stated that the destruction of superconductivity at the magnetic field orientation *φ* = 45° begins in fields significantly lower than at the orientations *φ* = 0° and 90°. A behavior of *R*(*T*) is caused by the internal magnetism of the Dy atoms which may strongly depend on the magnetic field orientation.

For synchrotron radiation sources in the X-ray range, which allow for the detailed observation of the structure of various materials (from biological objects, medical preparations, black rocks, to semiconductors), high-temperature superconductors based on oxide ceramics with a perovskite structure, such as YBa_2_Cu_3_O_7−*x*_ or Bi_2_Sr_2_CaCu_2_O_8+_*_x_* (where *x* is the degree of non-stoichiometry), are used. These materials have critical temperatures higher than 77 K (which allows the use of cheap liquid nitrogen for cooling) and can operate in magnetic fields up to 30 T.

Neutron sources use low-temperature superconductors (LTS) such as NbTi [33] and Nb_3_Sn [34] to create powerful magnetic fields that accelerate and focus particle beams onto a target, resulting in the production of neutrons. NbTi is an economical and technologically advanced material used in magnets with inductions up to 10–15 T; however, for higher fields (greater than 10 T), more advanced materials such as Nb_3_Sn are required, but their production is more difficult and expensive. These superconducting magnets are essential components of neutron sources, allowing the creation of strong magnetic fields without energy loss to control charged particles.

In [63], the methods of alloying and the geometry of composites that affect the structure of nanocrystalline layers of Nb_3_Sn in Nb/Cu-Sn superconducting composites are considered. Additionally, [43] investigates the structural parameters of the elements in the cables of technical superconductors based on the Nb-Ti alloy during multi-stage drawing.

The ISMAN—Institute of Structural Macrokinetics and Materials Science of the Russian Academy of Sciences is conducting active research on the production of high-temperature superconductors via the self-propagating high-temperature synthesis (SHS) method.

The method of time-resolved diffraction (TRXRD) was used [64] to study the effect of heating rate on phase formation during a thermal explosion in a helium atmosphere, with a mixture of Mg + 2B. It was shown that the MgB_2_ phase forms without intermediate compounds. An essential factor affecting the kinetics of MgB_2_ formation is the presence of oxygen impurities. At a heating rate of 150–200 °C/min, the oxide film on the surface of magnesium particles does not have time to form, which allows the reaction Mg + 2B = MgB_2_ to proceed via a reactive diffusion mechanism immediately after magnesium melts. The synthesis products consist mainly of MgB_2_ with traces of MgO at a level of 5%. The thermal explosion temperature is 1100 °C. At a heating rate of 30–50 °C/min, a comparatively thick oxide film forms on the surface of magnesium, which slows down the spreading of the melt and delays the onset of the MgB_2_ formation reaction by 8–9 s. The synthesis products contain MgB_2_ and up to 15% MgO, with the thermal explosion temperature being 1020 °C.

The production of materials from low-exothermic mixtures is a method of volumetric combustion in thermal explosion mode, which involves preheating the briquette to the self-ignition temperature, encompassing the entire process—especially when producing superconductors based on magnesium diboride [51,65,66].

The synthesis of the superconductor YBa_2_Cu_3_O_7−*x*_ (Y123) was carried out [62] using two different combustion synthesis modes: self-propagating high-temperature synthesis and thermal explosion. The influence of several processing variants, including the density of the raw materials, the oxygen pressure during synthesis, and the particle sizes of Cu and BaO_2_, on the formation of Y123 was investigated. The use of Cu particles ranging from <10 µm to 44 µm allowed for the regulation of the material synthesis process.

A new class of high-temperature superconductors [67] with layered structures based on iron, such as *RE*OFeAs (*RE* = La, Ce, Nd, Pr, Sm…), *A*Fe_2_As_2_ (*A* = BaS_2_…), *A*FeAs (*A* = Li…), and FeSe (Te), is characterized by distinctive features in phonon structures, spin waves, magnetic ordering, and Cooper pairing in these compounds.

In [52], calculating the AC losses in stacked high-temperature superconducting tapes requires the use of advanced finite element methods (FEM) or homogenization techniques to handle the complex electromagnetic interactions within the stack, as presented. While detailed modeling is accurate but computationally expensive, homogenization offers a faster approach by treating the tape stack as an anisotropic bulk material, allowing for significantly faster calculations without sacrificing accuracy in large-scale applications. Key factors affecting these losses include the power-law relationship between the electric field and current density, magnetic field amplitude, frequency, temperature, and the presence of ferromagnetic materials.

Superconducting ribbon coils and cables by Rebele [41] have also been modeled as stacks of parallel superconducting ribbons, each carrying the same transport current. The reduction in AC losses is achieved through proper scaling of the stack model containing 10–20 ribbons.

HTS materials YBaCu_2_O_5_ and Tl_1.5_BaCa_2_Cu_2.5_O_8_ were synthesized [53] by removing the barium copper oxide BaCuO_2_ and YBa_2_Cu_3_O_7_, as well as barium-tallium copper oxide BaCu_0.5_Tl_0.5_O_2_ from Tl_2_Ba_2_Ca_2_Cu_3_O_10._ It was found that these compounds contain less toxic Ba and Tl. The Meissner effect observed in these materials confirms their classification as HTS.

Superconducting YBCO compounds produced via ceramic technology and ceramics compacted from micro- and nanopowders with densities ranging from 2.6 to 5.8 g/cm^3^ and oxygen content of 6.83 were studied [54]. It was shown that an increase in sintering temperature leads to a reduction in the oxygen content in YBCO cells and changes their superconducting characteristics at *T_c_*.

The use of coated conductors [55] for levitational and trapped magnetic fields allows for the creation of magnetization loops in fields up to 14 T at temperatures ranging from 4.2 to 77.4 K. The levitation forces exhibited linear amplitudes and improved relaxation characteristics with an increased number of stacked ribbons.

A magnetic bearing design [68] based on YBa_2_Cu_3_O_7−*x*_ (YBCO) has been proposed, thanks to its ability to capture and hold the magnetic field over extended periods. However, this material requires reinforcement and has low thermal conductivity, which limits its practical applications.

In work [69], the suppression of levitation forces and magnetization between a NdFeB permanent magnet and a stack of HTS ribbons made of *h* = 5–100 *RE*BCO (12 × 12 mm) was demonstrated in both field and field-free cooling modes under crossed magnetic fields (up to 80 mT in the frequency range of 50 to 400 Hz).

A characteristic feature of the propagation of thermal instabilities [70] in technical superconductors under different cooling conditions is the abrupt transition from the superconducting to the normal state, with a subsequent temperature rise, characteristic of chain reactions.

A new method for calculating current paths [71] in HTS ribbons with various defect cracks and inclusions has been developed, allowing for the calculation of the spatial distribution of currents passing through a defective HTS ribbon.

It was established that, to form macrostates of superconducting ribbons [44], there are characteristic values of electric field intensity, which are influenced by factors such as current injection speed, superconducting properties, cooling conditions, and stabilizing matrix. These factors determine the volt-ampere characteristics of technical superconductors and help ensure the superconducting material’s temperature regime.

Superconductors made from MgB_2_ powders, which were subjected to pressing followed by sintering at 800 and 950 °C, were studied [45]. It was shown that, during sintering, the MgB_2_ phase dissolves in the formed liquid magnesium, leading to the formation of dense and porous regions in the material. To normalize the structure and homogeneity of the superconductor, the material is reprocessed.

In work [46], a comprehensive model for a ring-shaped HTS magnet was developed and its parameters were calculated, considering the transport characteristics, magnetic properties of HTS ribbons, and the layered structure of the material.

The influence of critical current density on levitation force from a stack of SC ribbons was established [72]. It was found that a higher critical current increases the levitation force, and with the number of layers ranging from 10 to 100, saturation of the levitation force is reached.

It was demonstrated [73] that magnetization of stacks of NdFeB permanent magnets, as a function of levitation force and the gap between the magnet and a stack of second-generation HTS ribbons GaBaCu_3_O_7_, depends on the cooling mode, calculated heat exchange, and the temperature distribution across the substrate and stabilizing layers Cu-Ag.

It was established that, during switching moments, with various current pulse amplitudes in homogeneous magnetic fields, there are [74] thermal bursts during the pulse at nonequilibrium states under current and magnetic field influence.

In modeling the magnetic system [75] of a contactless bearing made from HTS ribbons 2G, improved power characteristics comparable to those of HTS volumes in ribbons were observed.

Superconducting coatings (Fe/Se_1−*x*_Te*_x_*) were deposited on iron substrates (strip, ribbon) by vapor deposition or from molten halides. It was established that pure iron is the optimal substrate. At *T* = 4 K, the critical currents of the coatings were found to be 103 A/cm^2^ and showed minimal changes with increasing magnetic field [76].

In the study of thin disordered films of HTS based on YBCO, the theoretical possibility of forming an *s*-phase superconducting pairing in disordered *d*-type superconductors YBa_2_Cu_3_O_7−*x*_ was confirmed as the mean free path length decreases [77].

Based on the reviewed works, the most common technologies for obtaining high-temperature superconducting powders and materials from them include the following methods:

- Solid-phase reaction: Mixing the starting oxides or salts in a certain ratio, calcining (sintering) the mixture at high temperatures, and subsequent pressing and sintering of the material.
- Plasma spray: The starting materials are sprayed in a plasma stream and deposited as a thin layer on a substrate, yielding homogeneous powders with high dispersion.
- Oxidative synthesis: The starting materials undergo oxidation with an organic reagent (e.g., citrate or glycolate). This results in metal–organic complexes that decompose upon heating to produce oxide powders.
- Sol–gel method: The starting materials are dissolved in water or another solvent with the addition of a complexing agent (e.g., acetylacetonate or ethylene glycol). This forms a colloidal solution (sol), which transforms into a gel upon changes in pH or temperature. The gel is then dried and calcined to produce oxide powders.
- Spray drying: A solution of the starting materials is sprayed into a hot gas stream (air), causing rapid evaporation of the solvent and the formation of fine powder particles.
- Acetate method: The starting materials are dissolved in acetic acid with added ammonia or other bases. This forms metal acetates, which are then precipitated from the solution by changing the pH or concentration. The precipitate is dried and calcined to produce the starting powders.

Base HTS such as YBa_2_Cu_3_O_7−_*_x_*, Bi_2_Sr_2_CaCu_2_O_8+_*_x_*, Ti_2_Ba_2_CaCu_2_O_8+*x*_, and others have been produced using standard solid-phase reactions, plasma spraying, and oxidative synthesis. Superconducting magnetic systems for maglev (magnetic levitation) transport, including NbTi and Nb_3_Sn, are fabricated as multifilamentary composite superconductors using sol–gel and spray-drying methods. The synthesis of high-temperature superconductors based on barium, calcium, and complex systems like Bi–Sr–Ca–Cu–O and Ti–Ba–Ca–Cu–O generally involves using acetic acid as a solvent to form acetate salts, which are then sintered to produce high-dispersion oxide powders.

At the G. V. Kurdyumov Institute for Metal Physics (IMP) of the National Academy of Sciences of Ukraine and the Kyiv Academic University, research on superconducting materials is conducted under the guidance of academician NAS A.A. Kordyuk. The main research directions of the institute include the electronic structure of metals and alloys, atomic structure, the physics of strength of nanostructured systems, and more.

In particular, academician A.A. Kordyuk and co-authors [78,79] studied the electronic structure and properties of high-temperature superconductors (HTS) using angularly resolved photoemission spectroscopy (ARPES). They presented results on various types of HTS—cuprates, borides, and iron selenides. Possible mechanisms of superconductivity were proposed, and renormalization effects in HTS were studied using ARPES and other spectroscopic methods. These instruments provided energy spectra of electrons as a function of momentum and temperature. A comparative analysis of these spectra with theoretical calculations based on different electron-phonon and magnetic excitation interaction models was conducted.

Academician Kordyuk’s research has explored a wide range of superconductors and processes occurring in these materials under various experimental conditions. He places particular emphasis on studies of electronic structure, electronic and electrical properties, superconductivity mechanisms, electronic ordering of structure, vortex matter, surface physics, high-temperature cuprates, iron-based superconductors, amorphous superconductors, and dichalcogenides. The methods and applications involved include ARPES, tunneling spectroscopy, magnetization and transport, levitation-based methods.

ARPES is a method that allows for the direct observation of the Fermi surface and the underlying electronic structure of metals and metal oxides. These are the fundamental concepts for describing all electronic properties of solid materials and understanding key electronic interactions [80]. This technique is used in physics to study both macroscopic and microscopic physical properties of materials that arise due to electromagnetic forces between atoms, and it is particularly useful for investigating high-temperature superconductors at low temperatures.

At the B. I. Verkin Physics and Technology Institute of the National Academy of Sciences of Ukraine, fundamental research is being conducted in theoretical physics, mathematics, and applied physics. The main research directions include high-temperature superconductivity, weak superconductivity, magneto-optics of antiferromagnets, physics of low-dimensional systems, microcontact spectroscopy, quantum crystals, nonlinear phenomena in metals, and quantum phenomena in plasticity, among others [28].

Quasicrystalline high-temperature superconductors, treated with molten material, were studied using the resonance oscillation method [79]. It was found that in the amplitude dependence of alternating current losses on the magnetic field amplitude, sharp and well-aligned dynamic transitions are observed due to the absence of barriers at low field-changing speeds and the appearance of transitions at higher speeds due to phenomena associated with vortex propagation along the surface.

It has been noted that the key to producing high-quality superconductors is the fine-grained structure of their phase composition. Using Nb_3_Sn as an example, it is shown that the core of the material’s production involves a technology where niobium rods are initially fabricated inside a copper tube, which is then filled with tin. Hexagonal blanks (sub-elements) are placed into the copper shell and deformed to produce composite wire with a diameter of less than 1 mm, containing niobium fibers smaller than 1.5 microns. This technology increases the current density from 1000 A/mm^2^ to 2500 A/mm^2^ due to the fine-grained structure of the superconductor [2].

The work [81] is dedicated to studying the enhancement of linear gain in the gigahertz frequency range without using feedback circuits. The microwave amplifier based on a high-temperature SQUID includes identical and parallel-connected first and second Josephson contacts, formed in the HTS layer and placed along the bicrystalline boundary of the substrate. The input inductive element is included between the adjacent current leads of the Josephson contacts. Additionally, third and fourth Josephson contacts are introduced, with the critical current of the first and second contacts coinciding, while the thirds are smaller, and the fourths exceed the previous value. The HTS layer forms a track that crosses the bicrystalline boundary twice, creating a closed loop with the mentioned inductive element located on one side of the bicrystalline boundary. The third and fourth Josephson contacts are placed at the intersections of the track with the bicrystalline boundary, with the track width at the location of the fourth contact being larger than the corresponding width for the third contact.

Systematic studies of HTS films with excess Gd relative to the stoichiometric composition of GdBaCu_3_O_7_ [82] showed that when growing the film, thread-like defects in the form of a non-superconducting phase Ga_2_CuO_4_ appear along the *ab*-plane. This leads to the application of the vortex pinning mechanism and a peak in the critical current at +15% Gd.

In several earlier works [83,84], attention is drawn to the phenomena observed during SHS (Self-Propagating High-Temperature Synthesis) processes, particularly the dynamics of phase formation in HTS depending on the number of reagents used. Also, X-ray diffraction (*XRD*) data reveals the mechanisms of component interactions during solid-phase high-temperature combustion processes. Superconductors based on Y and Tl, produced using SHS technology, are characterized by a relatively high critical current density at 77 K and zero magnetic field, with the values can range from approximately 10^3^ A/cm^2^ for polycrystalline samples to over 10^6^ A/cm^2^ for the high-quality films or coated conductors in self-field conditions for YBa_2_Cu_3_O_7−_*_x_* [85] and a *J_c_* value for Tl_2_Ba_2_Ca_2_Cu_3_O*_x_* are 230 A/cm^2^ for bulk superconductors, up to 1.8 MA/cm^2^ for coated conductors and 2.8 MA/cm^2^ for films [86].

At the Institute of Combustion Problems of the Ministry of Science and Higher Education of the Republic of Kazakhstan, superconducting MgB_2_ powders were obtained from magnesium diboride synthesized by the solid-phase combustion (in other words like SHS) method under high-pressure conditions (25 atm) of an inert gas (argon) in a specially designed thick-walled steel reactor [87]. The research results showed that the high temperatures developed during SHS, and high pressure improve the characteristics of MgB_2_, namely: increasing the critical current density to 3.8 × 10^6^ A/cm^2^, and the value of superconducting volume fraction (SCF) that was equal to 16%. This value of SCF at a magnetic field of 10 Oe and 5 K indicates that the superconductivity is bulk in nature. These data indicate that there is a practical possibility of producing large-scale industrial products.

Studies have also been conducted to improve the superconducting properties of magnesium diboride by doping it with multi-walled carbon nanotubes (MWCT) (1%) [88]. A transition to the superconducting state in a bulk MgB_2_ with the addition of MWCT (1%) was observed at 38.5 *K* and zero magnetic fields, with a critical current density of 1.4 × 10^8^ A/cm^2^ at 5 K.

The use of graphite, single-walled, and multi-walled carbon nanotubes as doping additives significantly accelerates the synthesis process of MgB_2_ (2–5 min) while achieving good critical temperature values of 38.8–39.5 K and current density of 2.7 × 10^6^ A/cm^2^ at 5 K. Single-walled carbon nanotubes proved to be the most effective in doping the synthesized magnesium diboride [89].

The application of centrifugation [90] during the synthesis of MgB_2_ slightly increases the critical current density (*J_c_*) of the superconductor, while the critical transition temperature remains unchanged at 37.5–38.0 K.

A critical transition temperature of over 39.5 K was achieved [91] by doping MgB_2_ with barium oxide (in a bulk form), resulting in a critical current density of 1.7 × 10^6^ A/cm^2^ due to the formation of a new MgB_2_ phase with an increased number of effective pinning centers.

In the production of superconducting composites based on YBa_2_Cu_3_O_7-8_ (YBCO) [92], the authors employed a synthesis technology based on optimizing the time and conditions of thermal treatment. Additionally, chemically doping the material with microparticles of Al, Ni, and Fe significantly influenced the formation of the superconducting Y_123_ phase.

In the article by Dautova L. M. et al. [93], calculations and simple analytical formulas for the critical transition temperature, *T_c_* and the Coulomb pseudopotential m* are presented, which allow the calculation of the constant *I* and I* = I − m*. The approach used to determine m* is supported by the phenomenon of tunneling effects in quantum mechanics and the “thermal pinning” observed in experiments on scattering slow particles in nuclear physics.

According to the method developed by Karaganda University, named after E. A. Buketov [94], to reduce the width of the transition to the superconducting state and increase the critical transition temperature of YBaCuO films, the substrate temperature of SrTiO_3_ should be maintained at (1093 ± 1) K, and the pressure in the working chamber should be 1.31 × 10^−3^ Pa.

This paper [63] presents the results of obtaining copper salts using organic acids, in particular, the production of divalent copper formates, which can be used as an inexpensive component for the synthesis of copper-doped alkaline earth superconductors and high-temperature superconductors based on cuprates. The method for producing copper(II) formate involves mixing copper nitrate and formic acid, followed by holding and cooling, wherein the initial reagents are taken in the ratio Cu(NO_3_)_2_⋅3H_2_O: (HCOOH) = 1 ÷ 2.5 − 1 ÷ 3.0 at a formic acid concentration in the range of 20–99.7% and mixing is carried out at room temperature with holding at this temperature for 10–15 min or with subsequent addition of water in an amount of 10–12 wt.% of the total mass and heating to 80 °C with holding at this temperature for 15–20 min and cooling again to room temperature, or at a temperature of 50 °C with subsequent evaporation for 90–95 min and cooling to room temperature. It has been shown that the cuprate-based superconductors synthesized using this technology exhibit a uniform phase composition—components throughout the material’s volume properly [95].

Experimental data on a new class of high-temperature superconductors [62] and their layered structure in iron-based compounds *RE*OFeAs, where *RE* = La, Ce, Nd, Pr, Sm, etc., *A*Fe_2_As_2_ (*A* = Ba, Sr, …) *A*FeAs (*A* = Li, …) and FeSe(Te) are presented. The study of their electronic spectrum structure highlights the role of correlations, spectra, and collective excitations (photons, spin waves). Key models have been developed to describe the existence of possible types of magnetic ordering and Cooper pairing in these compounds.

One noteworthy fact is that, in the 1970s and 1980s, technical superconductors were produced in Kazakhstan for the TOKAMAK-7 in 1978 and TOKAMAK-15 in 1988 [96]. Currently, the production of superconductors is organized at the Chepetsky Mechanical Plant (ChMZ) in Glazov for the magnetic system of the International Thermonuclear Experimental Reactor (ITER) project. More than 200 tons of superconducting materials have been produced for this project using this technology [97].

Superconducting quantum interferometers (SQUIDs) based on iron-nitrogen compounds, with a resolution of 10^−6^ *Φ*_0_/Hz ^1/2^ = 2.07 *×* 10^−21^ *Bb*^1/2^, are connected to a magnetometer and function as a tensor-magnetic converter for measuring pressure with a sensitivity of 10^−13^ Pa/Hz^1/2^ and measuring relative elongation with a sensitivity of 10^−24^ Hz^1/2^ [98]. These systems enhance the accuracy of determining the mechanical properties of materials and can also be used as receivers for gravitational waves.

## 5. Promising Directions in Superconductor Research

The Physics Department of Lomonosov Moscow State University has a long tradition in superconductivity research, especially in superconducting electronics. In the late 1980*s*, MSU scientists pioneered the concept of dynamic single-flux-quantum logic (also known as RSFQ logic) for digital superconductor circuits—a breakthrough in high-speed, low-energy logic that was later universally adopted. Today, MSU’s researchers continue both theoretical and experimental work on superconductivity and superconducting electronics. For example, the Laboratory of Nanostructure Physics at MSU studies hybrid nanostructures containing superconducting materials, designing novel device architectures (including superconducting analog/digital circuits and neural networks) based on Josephson junctions and superconducting spintronics [47].

National Research Centre Kurchatov Institute is conducting intensive studies [17] and testing superconductors for future collider applications based on niobium-titanium superconductors at low temperatures and magnetic fields of varying strengths. The Future Circular Collider (FCC)—a massive particle collider with a circumference of up to 100 km—will require three thousand tons of superconducting wires based on Nb-Ti alloys. Superconducting materials for accelerators are substances that exhibit zero electrical resistance at a certain temperature and are capable of generating strong magnetic fields to control the motion of charged particles in accelerators. Low-temperature superconductors based on NbTi or Nb_3_Sn alloys with critical temperatures of 10 K and 18 K and magnetic fields of 10 T and 2 T are used in colliders.

The intensity [48] of the trapped field in HTS based on a ribbon stack (*RE*)VSO (12 × 12 mm) at temperatures ranging from 4 to 80 K and in constant magnetic fields up to 8 T showed a nonlinear character with a tendency to saturate at *n* > 60, exceeding 2.5 T at *T* = 4 K.

One of the significant achievements is the prediction of the existence of thermionic phenomena in superconductors, based on which the phenomenological theory of superconductivity and superfluidity of liquid helium by V. L. Ginzburg was established [38].

The Ginzburg-Landau phenomenological theory allows one to obtain an accurate result without establishing the true causes of the phenomenon of superconductivity of the material and does not consider their internal mechanisms [99,100]. One of the important consequences of the Ginzburg-Landau theory was the discovery of Abrikosov vortices in type II superconductors located in a strong magnetic field [101].

Notably, the explanation of the superconductivity mechanism emerged with the development of the Bardeen-Cooper-Schrieffer (BCS) theory [49], which is based on the concept of the Cooper pair. This pair represents a correlated state of electrons with opposite spins and momenta. In 1972, the creators were awarded the Nobel Prize in Physics. L. Cooper pointed out the possibility of forming a bound state of two electrons at the Fermi level during the exchange of phonons, which can be considered as the interaction of electrons with the ionic crystal lattice. An electron attracts ions and creates a positive charge density, which attracts another electron, opposite in spin charge and velocity [100].

The study of the mechanism of Cooper pair formation and phase transitions in superconductors is the focus of research at the L. D. Landau Institute for Theoretical Physics of the Russian Academy of Sciences. It should be noted that the Institute is one of the leading scientific centers in Russia, engaged in research in the fields of condensed matter physics, quantum field theory, relativistic astrophysics and cosmology, quantum computing, and mathematical physics [102]. The primary mechanism for the formation of superconductivity, as previously demonstrated, involves Cooper pairs. These are weakly bound pairs of electrons that move through the material’s lattice without scattering off its atoms [103]. In contrast, this is an electron-phonon phenomenon, arising from the interaction during the exchange of energy momentum between electrons and lattice phonon vibrations, leading to an effective attraction of electrons and the formation of a coherent quantum state (Bose-Einstein condensate) [104].

It has been shown that the transition of a material from a normal to a superconducting state is accompanied by phase transitions at a specific critical temperature, *T_c,_* and depends on the composition and crystalline structure of the substance. The phase transition is accompanied by changes in the thermodynamic parameters of the material, such as entropy, specific heat capacity, magnetic susceptibility, and other properties. The phase transitions of superconductors are described by the well-known Ginzburg-Landau model, which uses the wave function of Cooper pairs. This theory allows for the classification of superconductors by type and their behavior in a magnetic field [105].

The work of young scientists [106] from the Ust-Kamenogorsk Technical University on the study of superconducting materials for magnetic resonance imaging (MRI) demonstrated that these materials can carry powerful electric charges without heating up. A prototype superconducting wire made of titanium and niobium was manufactured at JSC “Ulba Metallurgical Plant.”

At the Toraighyrov University in Pavlodar [107], a study was conducted in which Kazakh researchers established the influence of a nanoscale additive on the structure of high-temperature superconducting materials on the critical temperature, with a significant increase in the superconducting current density. The critical transport current density J*_ct_* was measured using the nickel-zinc ferrite criterion Ni_0.5_Zn_0.5_Fe_2_O_4_(NZFO) on the Bi_1.6_Pb_0.4_Sr_2_Ca_2_Cu_3_O_10_ (Bi-2223) superconductor. To create additional pinning centers, nanoscale impurities Co_0.5_Zn_0.5_Fe_3_O_4_ and Ni_0.5_Zn_0.5_Fe_3_O_4_ were used with a mass fraction of 0.1%.

To obtain high-temperature superconductors, a patent [58] was granted at the Institute of Combustion Problems of the Ministry of Education and Science of the Republic of Kazakhstan for the technology of manufacturing thick and thin superconductors. The patent showed that when making strontium bismuth cuprate HTS, a mixture of R_2_PbH_2_ and one of the metals Sr, Ca (0.1–1.0 mass%) is used.

Researchers [59,60] from the National Academy of Sciences of the Republic of Armenia, particularly in the works of Tataryan A.A., demonstrated that by using various polymer binders, superconducting polymer-ceramic nanocomposites are obtained, in which the critical transition temperatures to the superconducting state are 1–3 K higher than those of the original ceramics. It was shown that by co-polymerizing metal monomers with YBa_2_Cu_3_O_7−_*_x_*, they acquire superconducting properties.

Osipyan Institute of Solid-State Physics RAS is a center for experimental research at low temperatures, strong magnetic fields and high pressures. One of the areas worth noting is the study of superconductivity in amorphous, nanocrystalline and composite materials [20].

In the works of Jumonov Sh. S. [18] from the National Academy of Sciences of the Republic of Uzbekistan, ultra-doped cuprates LSCO and Bi2212 based on large polarons and modified BCS-like models of precursor pairing of polar carriers above T_c_ were studied. The possibility of forming (unpaired and paired) types of pseudogaps was demonstrated, and phase diagrams of the normal states of HTS cuprates LSCO and Bi-2212 were constructed, as well as the possibility of their existence at temperatures T_r_ ≈ T* above *T*_c_. It was established that these processes correspond to transitions from the metallic state to the pseudogap state (at *T = Tr*) and from the polar pseudogap state to the BCS-like pseudogap state (at T = T*).

A conference dedicated to the current state of research in the development and production of superconducting materials [19,35] was attended by participants from Mordovian State University named after N. P. Ogarev, the Andronikashvili Institute of Physics of the Georgian Academy of Sciences, the Institute of Nuclear Physics of the Academy of Sciences of Uzbekistan, the Physico-Technical Institute of the Academy of Sciences of Uzbekistan, and the Ministry of Energy of Uzbekistan. Research by Uzbek scientists confirmed that the formation of high-temperature superconductivity (HTS) using Large Solar Energy allows the production of stable superconducting compositions with high critical transition temperatures. A broad peak (Δ*T*~100 K) with a maximum at *T* ≈ 200 was detected in low-frequency dynamic experiments in the study of magnetic properties of multiphase cuprate superconductors Bi_1.7_Pb_0.3_Sr_2_Ca_n−1_Cu*_n_*O*_y_* (*n* = 2–30), synthesized using solar energy and ultrafast melt quenching [36].

Scientists from the Republic of Moldova [22], after the discovery of superconductors based on MgB_2_ (*T_c_* = 40 K), found that some of their properties with two energy bands were theoretically reconsidered in a series of their studies on diborides and borocarbides. These works contain their developed key physical concepts based on the equations of thermodynamic and electromagnetic characteristics for this type of superconductors. Research on the properties of two-band and multi-band superconductors, carried out under the guidance of V. A. Moskalenko [23], revealed a new direction in low-temperature physics—the creation of superconductors with an anisotropic energy spectrum. It was noted that the properties of superconductors with overlapping energy bands differ significantly from single-band superconductors in both quantitative and qualitative terms.

Physicists from Azerbaijan [108] are actively involved in priority mega-science projects and in preparing experiments on the NICA (Nuclotron-based Ion Colliderer &Actility) superconducting proton and heavy-ion collider at the High-Energy Physics Laboratory (HEPL) of the Joint Institute for Nuclear Research (JINR) in Dubna.

The National Academy of Sciences of Belarus (NASB) is an umbrella organization comprising over 100 research institutes and centers across various scientific domains. Citing the NASB as a whole for a specific topic like superconductivity is therefore too broad—it is important to identify the institutes involved. In Belarus, superconductivity research is concentrated in a few key institutions. Within NASB, the primary center for superconductivity studies is the Scientific-Practical Center for Materials Science of NAS Belarus. Additionally, several leading university-based institutes carry out superconductivity research, notably the National Center for Particle and High Energy Physics of Belarusian State University (BSU), the Research Institute for Nuclear Problems of BSU, and the Belarusian State University of Informatics and Radioelectronics (BSUIR). Below, we provide an overview of these institutions and the superconductivity research conducted there, along with references to their specific activities and contributions [24].

Scientists and specialists [25] from the Republic of Belarus developed, manufactured, and tested three experimental samples of a superconducting niobium resonator of the electrical type at a temperature of liquid helium (4.2 K) for accelerators of electrons and positrons, with a frequency of 1.3 GHz.

In line with the initiative of the Republic of Belarus [26], considering the high interest from CIS countries in deepening scientific and technical cooperation, on 31 May 2019, the Council of Government Members of the CIS adopted a decision to develop an Intergovernmental Program for Innovative Cooperation among the CIS member states until 2030. Belarus, together with partners from the Commonwealth, developed the concept of the Program. The implementation of this program will contribute to the development and introduction of unique models of new equipment and technology [109].

The Research Institute for Nuclear Problems (INP) of BSU is another leading scientific institute that engages in superconductivity research, especially in applied contexts. In recent years, INP BSU took a lead role in building and testing a niobium superconducting radio-frequency resonator for particle accelerators. In December 2022, for example, INP scientists reported the successful low-temperature tests of a home-grown niobium resonator, during which they observed its phase transition into the superconducting state [110].

Research on high-temperature superconductors [111] showed that the materials consist of a complex electronic structure, and the superconducting state is preceded by the presence of a “pseudogap phase.” In the superconducting state, electrons in the material form pairs near a specific level—the Fermi level, which is separated by a special gap. This energy interval exists between levels filled with identical electrons located beneath the Fermi surface. Since the gap forms even before the transition to the superconducting state, it is called the “pseudogap.” At the Keldysh Institute of Applied Mathematics of the Russian Academy of Sciences, physicist V. L. Lakhno suggested that the theory of the “pseudogap phase” is based on a translationally invariant bipolar mechanism of quasiparticles (bipolarons). It was established that the pseudogap phase exists above the critical temperature of the superconducting transition. The nature of the pseudogap in HTS is associated with the existence of paired electronic states for *T* > *T_c_* (TI bipolarons).

The pseudogap [78] is a reduction in the electronic density at the Fermi level, observed in many systems. However, since the discovery of HTS (1986), this phenomenon was initially attributed exclusively to high-temperature superconductors until two distinct energy scales were later identified in the form of the “two-gap scenario” and charge density wave models in cuprates. According to results from angle-resolved photoemission spectroscopy (ARPES), this is a complex phenomenon involving three “interwoven” orders: spin and charge density waves, along with preformed pairs that emerge in different parts of the phase diagram. It has been established that density waves in cuprates compete with superconductivity for electronic states but, at the same time, bring the electronic structure closer to a Lifshitz transition, leading to similarities between superconductors based on cuprates and iron-based materials.

It has been determined [112] that the electronic structure of pnictides differs significantly from that of cuprates. However, these differences suggest the existence of a common “coarse” mechanism that does not depend on the fine details of the band structure and is responsible for superconducting pairing in these materials. The authors propose a qualitative model describing the ground state and the mechanism of superconducting pairing in cuprates and pnictides. This model may potentially explain many of their unusual properties.

Using neutron spectroscopy of crystal electric field (CEF) levels [113], a model has been proposed [111] that includes a mechanism for generating additional free carriers in cuprates and pnictides during heterovalent and isovalent doping. This model allows for determining the precise position of «122» pnictides and predicting their sign, which in some cases does not match the sign of the doped carriers. It was previously shown [2] that most anomalies in the superconducting properties of cuprates and iron-based pnictides, observed within the superconducting dome and its position on phase diagrams, do not require knowledge of the electronic structure. They can be calculated and understood within the framework of the proposed model, which describes the cluster structure of the superconducting phase and assumes self-localization of doped carriers, where each doped carrier locally distorts the electronic structure.

To understand the pseudogap state in HTS, experimental findings and theoretical models were analyzed in, demonstrating that fluctuations of ferromagnetic order and their interaction with charge carriers lead to an anisotropic renormalization of the electronic spectrum in the form of a non-Fermi liquid at specific regions of the Fermi surface.

Maximov D. G. et al. [114] discussed possible mechanisms of HTS. The authors identify various electron interactions in cuprates, highlighting the significant role of electron-phonon interaction.

The structure of the magnetic flux [115] on the surface of single crystals of EuFe_2_(As_1−*x*_P*_x_*)_2_, doping with phosphorus (*x* = 0.20 and 0.21), was investigated. The authors established the vortex structure of the frozen magnetic flux at *T_c_* = 22 K and ferromagnetic *T_c_* = 18 ± 03 K, along with phase transitions. It was shown that below the temperature of the ferromagnetic transition, a magnetic domain structure was observed in the superconducting state [116].

Results from the application of angle-resolved photoemission spectroscopy (ARPES) with temperature-dependent angular resolution for investigating various iron-based superconductors (FeSe, Fe(Se, Tl), Ba(Co)) are presented [56]. These studies allow for the evaluation of important parameters needed to understand the superconductivity processes.

Ukrainian scientists [57] have developed a simple-to-manufacture thin-film superconducting quantum interferometer (SQI) with ultra-low inductance (~10^−13^ Hz). Using the SQI, the magnetic field penetration depth in a 50% In and 50% Sn alloy film was determined for the first time.

A SQUID sensor [50] has been experimentally implemented with a sensitivity of 0.26 µA/*Φ*_0_ based on tunneling junctions in an Nb/AlO*_x_*/Nb material, showing low sensitivity to external fields and electrostatic interference.

This review of materials published in scientific literature and other informational sources reveals that a number of studies in the CIS continue at the academic level in certain areas. Special mention should be made of the significant contribution of scientists from the Russian Federation, particularly in the fields of solid-state physics and HTS materials. The fragmentation of research on superconducting materials in the former Soviet republics suggests the need for the unification of efforts, increased contacts, and initiatives from the heads of CIS to develop joint documents for cooperation in fundamental research of promising directions. In particular, the concept of cooperation within the CIS in fundamental research, including superconducting materials, has significant potential and prospects. It is noted that one of the main steps in this direction is the support and encouragement of contact between scientific research centers of CIS, the organization of joint targeted research, the modeling of know-how projects in various sectors of the economy, the exchange of experience, and the training of specialists [26,109].

## 6. Conclusions

The conclusions of this study are as follows:

An overview of research on superconducting materials has been provided, including brief annotations of published papers and scientific cooperation among CIS countries (Armenia, Azerbaijan, Belarus, Kazakhstan, Moldova, Russia, Ukraine, Uzbekistan). It is shown that fundamental research on superconducting materials is being funded for development and study at the government level in each republic.

- The greatest contribution to superconducting research comes from scientists in the Russian Federation, particularly from the leading institutions of the Russian Academy of Sciences, such as the P. N. Lebedev Physical Institute (FIAN), A. F. Ioffe Physical-Technical Institute (IPTI), the L. D. Landau Institute for Theoretical Physics (ITF), and the I. A. Osepyan Institute of Solid-State Physics (ISSP).
- At FIAN, under the leadership of Academician V. L. Ginzburg, research on various metal compounds, alloys, ceramic materials based on Nb, Sn, Ge, La, cuprates, iron-containing pnictides, chalcogenides with lanthanides and actinides, and other metals was conducted.
- Research at the A. F. Ioffe Physical-Technical Institute was focused on developing thin superconducting films for radiation detectors, filters, etc. For example, the researchers created superconducting iron-containing membranes in which the critical temperature (T_c_) of the FeSe material increased from about 8 K at atmospheric pressure to about 37 K at 9 GPa, accompanied by magnetic ordering. This significant increase in *T_c_* under pressure is a known phenomenon for FeSe, with the highest *T*_c_ values observed in thin films or intercalated forms, classifying it as a medium-temperature superconductor.
- The ISSP RAS developed the phenomenological theory of superconductivity and superfluidity, which allows for accurate research results without necessarily explaining the true causes of the phenomena (Nobel Prize-winning theory by Landau and Ginzburg). This theory describes phase transitions involving changes in thermodynamic parameters using the Cooper pair wave function. In 2003, the Nobel Prize in Physics was awarded to A. Abrikosov, V. Ginzburg, and Anthony Leggett for their contributions to the understanding of superconductivity and superfluidity.
- Significant contributions to superconducting research were made by the National Research Nuclear University, with work by Dr. M. I. Yeremets on lanthanum (La, Y)H_6_ and decahydrides (LaY)H_10_ with a maximum critical temperature *T_c_* ≈ 253 *K*, magnetic field B_0_ ≈ 13.5 T at 183 GPa, and current density 12–27.7 kA/mm^2^, comparable to NbTa and NbSn at 4.2 K.
- At the L. D. Landau Institute for Theoretical Physics, the main mechanism of superconductivity was found to be the formation of Cooper pairs—weakly bound electron pairs that move without scattering on the atoms of the material’s lattice (electron–phonon interaction in the lattice).
- Publications from Ukraine, Kazakhstan, Belarus, Turkmenistan, Armenia, and Moldova, as well as collaborative works with Russian institutions, demonstrate high-quality research using modern methods and equipment, including techniques for creating low temperatures and producing highly purified components from pure metals, cuprates, iron-containing compounds with rare-earth elements, and other materials.
- It was established that high-temperature superconducting properties are improved when using high-purity materials, high synthesis temperatures, including self-propagating high-temperature synthesis (SHS) methods for preparing film-structured substances using lasers, deposition of high-dispersion nanoscale compounds from gas phases, and other techniques.
- Based on the presented research materials, it is concluded that a promising direction is the production of ultrathin film-structured materials on thin substrates with special properties and the development of technologies for their use. The application of additives in known cuprate superconductors, which form crystalline structures capable of improving superconducting properties and magnetic characteristics, is a significant development. The creation of new technological principles for material production using new methods, such as blending of precursor materials, maintaining the fractional chemical composition, pressing, and spinning under controlled thermal regimes, is also highlighted as a key area of future work. The development and enhancement of SHS technology for new material synthesis remains a priority.

## Figures and Tables

**Table 1:** Summary of Superconductivity Research in CIS Countries.

Country	Key Institutions/Research Centers	Research Focus	Notable Materials Studied	Significant Achievements/Findings	References
Armenia	National Academy of Sciences of Armenia	Polymer-ceramic superconducting nanocomposites	YBCO composites	Improved *T_c_* by copolymerization with metals	[18,19]
Azerbaijan	High-Energy Physics Lab (JINR, Dubna)	Superconducting systems for colliders	Proton and heavy-ion superconducting systems	Participation in NICA collider project	[20]
Belarus	National Academy of Sciences of Belarus, Scientific and Practical Center of the National Academy of Sciences of Belarus for Materials Science. National Center for Particle and High Energy Physics of Belarus State University, the Research Institute for Nuclear Problems of BSU, the Belarusian State University of Informatics and Radioelectronics.	Application in medical equipment, superconducting resonators	Nb-based superconductors	Development of niobium resonators for MRI and accelerators	[21,22,23,24,25,26]
Kazakhstan	Institute of Combustion Problems, Toraighyrov University, Karaganda University, Ulba Metallurgical Plant	MgB_2_ synthesis via SHS, doping effects, HTS films and composites	MgB_2_, YBCO, Bi-2223, NZFO-doped superconductors	High *J*_c_ values from CNT/MgB_2_, SHS optimization, substrate temperature effects on *T_c_*	[27,28,29,30,31,32,33,34]
Moldova	Institute of Applied Physics	Multi-band superconductors, anisotropic properties	MgB_2_, borocarbides	Theoretical modeling of anisotropic spectra	[35,36]
Russia	P. N. Lebedev Physical Institute, Ioffe Institute, ISMAN, L. D. Landau Institute, Kurchatov Institute, the Physics Department of Moscow State University, the Moscow Institute of Physics and Technology, the University of Science and Technology MISIS, the Moscow Aviation Institute. Nuclear University “MEPhI”	HTS and LTS synthesis, BCS theory, phase transitions, hydrides, SHS, pseudogap physics	Nb_3_Sn, MgB_2_, YBCO, Tl-based cuprates, La-Y hydrides, FeN_4_H_4_	Hydrides with *T_c_* up to 253 K, SHS of YBCO, pseudogap theory, Abrikosov vortices, MgB_2_ synthesis via SHS	[15,24,37,38,39,40,41,42,43,44,45,46,47,48,49,50]
Ukraine	G. V. Kurdyumov Institute for Metal Physics (IMP), Kyiv Academic University, B. I. Verkin Institute	Electronic structure, HTS mechanisms, vortex matter, amorphous and iron-based superconductors	Cuprates, borides, FeSe, In-Sn alloy, amorphous superconductors	ARPES studies of HTS, SQI with ultra-low inductance, electronic structure mapping, vortex dynamics, levitation methods	[16,50,51,52,53,54,55,56,57]
Uzbekistan	National Academy of Sciences of Uzbekistan, Physico-Technical Institute	Doped cuprates, polarons, solar furnace synthesis	LSCO, Bi-2212	Formation of pseudogaps, HTS synthesis via solar furnace	[58,59,60]

## Data Availability

No new data were created or analyzed in this study. Data sharing is not applicable to this article.

## Abbreviations

The following abbreviations are used in this manuscript:

CIS	Commonwealth of Independent States
HTS	High-temperature superconductors
LTS	Low-temperature superconductors
MWCT	Multi-walled carbon nanotubes
AFM	Antiferromagnetic
ARPES	Angularly resolved photoemission spectroscopy
SQUID	Superconducting quantum interference device
SHS	Self-Propagating High-Temperature Synthesis
XRD	X-ray diffraction
TRXRD	Time-resolved X-ray diffraction
BCS	Bardeen-Cooper-Schrieffer
NICA	Nuclotron-based Ion Colliderer &Actility
CEF	Crystal electric field
SQI	Superconducting quantum interferometer
FCC	Future Circular Collider

## References

[1] Nobuya B. (2023). Low-temperature superconductors: Nb_3_Sn, Nb_3_Al, and NbTi. Superconductivity.

[2] Sadovskii M.V. (2001). Pseudogap in High-Temperature Superconductors. Physics-Uspekhi.

[3] Poole C.P., Farach H.A., Creswick R.J., Prozorov R. (2010). Superconductivity.

[4] Larbalestier D., Gurevich A., Feldmann D.M., Polyanskii A. (2001). High-Tc Superconducting Materials for Electric Power Applications. Nature.

[5] Malozemoff A.P., Fleshler S., Rupich M., Thieme C., Li X., Zhang W., Otto A., Maguire J., Folts D., Yuan J. (2008). Progress in High Temperature Superconductor Coated Conductors and Their Applications. Supercond. Sci. Technol..

[6] Pęczkowski P., Zachariasz P., Zalecki R., Piętosa J., Michalik J.M., Jastrzębski C., Ziętala M., Zając M., Gondek L. (2024). Influence of polyurethane skeleton on structural and superconducting properties of Y-123 foams. J. Eur. Ceram. Soc..

[7] Prikhna T., Sokolovsky V., Moshchil V. (2024). Bulk MgB_2_ Superconducting Materials: Technology, Properties, and Applications. Materials.

[8] Goodenough J.B. Meissner Effect—An Overview|ScienceDirect Topics. https://www.sciencedirect.com/topics/chemistry/meissner-effect?utm_source.

[9] Zhizn R. Journal N. and A New Class of High-Temperature Superconductors—Now at the FIAN. http://www.nkj.ru/archive/articles/20235.

[10] Mitsen K.V., Ivanenko O.M. (2004). Phase Diagram of La_2–*x*_*M_x_*CuO_4_ as the Key to Understanding the Nature of High-*T*_c_ Superconductors. Physics-Uspekhi.

[11] Kopaev Y.V. (2002). High-Temperature Superconductivity Models. Physics-Uspekhi.

[12] Aksenov V.L. (2002). Neutron Scattering by Cuprate High-Temperature Superconductors. Physics-Uspekhi.

[13] Elesin V.F., Kapaev V.V., Kopaev Y.V. (2004). Coexistence of Ferromagnetism and Nonuniform Superconductivity. Physics-Uspekhi.

[14] Volkov E.P., Vysotsky V.S., Firsov V.P. (2012). First Russian Long Length HTS Power Cable. Phys. C Supercond. Its Appl..

[15] Bondarenko S.I., Guo Q., Fan J.D., Sivakov A.G., Krevsun A.V., Link S.I. (2018). Microscopic Study of the YBa_2_Cu_3_O_7−*x*_ Crystal. Mod. Phys. Lett. B.

[16] Information Card. https://is.ncste.kz/icard/view/12289.

[17] JINR. https://www.jinr.ru/about-en/.

[18] Academy of Sciences of the Republic of Uzbekistan. https://www.academy.uz/en.

[19] Ergashev I.A., AN RU, Tashkent (Uzbekistan). Inst. Yadernoj Fiziki (2006). Acoustic Relaxations in High-Temperature Superconductor YBa2Cu3O7-x.

[20] Heisenberg W. (1967). The role of phenomenological theories in the system of theoretical physics. Adv. Phys. Sci..

[21] Abrikosov A.A. (1957). QMC Copier. On the Theory of Superconductivity. J. Expti. Theoret. Phys..

[22] Moskalenko V.A. (2013). Priority of Moldovan physicists in the creation and development of the multiband theory of superconductivity. Electron. Process. Mater..

[23] Budagov Y.A., Trubnikov G.V., Shirkov G.D., Baturitsky M.A., Bogdanovich M.V., Kurochkin Y.A., Zalessky V.G., Kilin S.Y., Azaryan N.S. (2022). Cooperation of JINR with scientific institutions of the Republic of Belarus in the field of superconducting accelerator resonators. Phys. Elem. Part. At. Nucl..

[24] National Academy of Sciences of Belarus. https://milex.belexpo.by/o-vystavke/spisok-uchastnikov/natsionalnaya-akademiya-nauk-belarus.html.

[25] Priorities of International Scientific, Technical and Innovative Cooperation of the Republic of Belarus|SCIENCE AND INNOVATIONS—Scientific and Practical Journal. https://innosfera.by/node/5159.

[26] Concept of Turkmenistan’s chairmanship in the Commonwealth of Independent States in 2019. Proceedings of the 2019 Results of the Meeting of the Council of Heads of Government of the CIS and the list of adopted documents.

[27] Belyavsky V.I., Kopaev Y.V., Tuan N.N. (2008). Superconductivity in Cuprate Homological Series: Interlayer Dielectric Coupling of Superconducting Pairs. JETP Lett..

[28] Eglitis R.I., Kotomin E.A., Popov A.I., Kruchinin S.P., Jia R. (2022). Comparative ab initio calculations of SrTiO_3_, BaTiO_3_, PbTiO_3_, and SrZrO_3_ (001) and (111) surfaces as well as oxygen vacancies. Low Temp. Phys..

[29] Kuzmicheva T.E., Kuzmichev S.A. (2021). Pnictides of the AFeAs Family (A = Li, Na) Based on Alkali Metals: Current State of Research on Electronic and Superconducting Properties (Mini-Review). ResearchGate.

[30] Kuzmichev S., Kuzmicheva T., Morozov I., Boltalin A., Shilov A. (2022). Multiple Andreev reflections effect spectroscopy of LiFeAs single crystals: Three superconducting order parameters and their temperature evolution. SN Appl. Sci..

[31] Pavlov N.S., Shein I.R., Pervakov K.S., Nekrasov I.A. (2023). Electronic Structure of InCo_2_As_2_ and KInCo_4_As_4_: LDA + DMFT. Pisʹma V Ž. Êksperimentalʹnoj Teor. Fiz..

[32] Terekhov A.V., Zolochevskii I.V., Khristenko E.V., Ishchenko L.A., Bezuglyi E.V., Zaleski A., Khlybov E.P., Lachenkov S.A. (2016). Anisotropy of electric resistance and upper critical field in magnetic superconductor Dy_0.6_Y_0.4_Rh_3.85_Ru_0.15_B_4_. Phys. C Supercond. Its Appl..

[33] Cardwell D.A., Larbalestier D.C., Braginski A.I. (2022). Handbook of Superconductivity: Processing and Cryogenics, Volume Two.

[34] Zlobin A.V., Schoerling D., Schoerling D., Zlobin A.V. (2019). Superconducting Magnets for Accelerators. Nb3Sn Accelerator Magnets: Designs, Technologies and Performance.

[35] Director of Nuclear Research Institute: Azerbaijani Scientists Take Part in Such Priority Projects as NICA Collider—INTERVIEW. https://report.az/en/education-and-science/director-of-nuclear-research-institute-azerbaijani-scientists-take-part-in-such-priority-projects-as-nica-collider-interview.

[36] Chigvinadze J.G., Acrivos J.V., Ashimov S.M., Gulamova D.D., Donadze G. (2017). Superconductivity at T=200K in Bismuth Cuprates Synthesized Using Solar Energy. arXiv.

[37] Bezotosnyi P.I., Dmitrieva K.A. (2021). Modeling of the Critical State of Layered Superconducting Structures with Inhomogeneous Layers. Phys. Solid State.

[38] Ginzburg V.L. (2004). On Superconductivity and Superfluidity (What I Have and Have Not Managed to Do), as Well as on the “physical Minimum” at the Beginning of the XXI Century (December 8, 2003). Physics-Uspekhi.

[39] Ginzburg V.L., Andryushin E.A. (2004). Superconductivity.

[40] The Nobel Prize in Physics 2003. https://www.nobelprize.org/prizes/physics/2003/summary.

[41] Gerbshtein Y.M., Nikulin E.I. (2012). High-Temperature Superconductors YBaCu_2_O_5_ and Tl_1.5_BaCa_2_Cu_2.5_O_8_ with a Decreased Content of Heavy Metals (Ba and Tl). Phys. Solid State.

[42] Trojan I.A., Semenok D.V., Ivanova A.G., Kvashnin A.G., Zhou D., Sadakov A.V., Sobolevsky O.A., Pudalov V.M., Lyubutin I.S., Oganov A.R. (2022). High-Temperature Superconductivity in Hydrides. Physics-Uspekhi.

[43] Schlyakhova G.V., Barannikova S.A., Zuev L.B. (2015). The study of nanostructural elements of superconductive cable Nb-Ti. Izv. Ferr. Metall..

[44] Kuznetsova E.I., Degtyarev M.V., Blinova Y.V., Sudareva S.V., Aksentsev Y.N., Pilyugin V.P. (2017). Mechanism of Structure Formation during High-Temperature Annealing of Pressure-Deformed Bulk MgB_2_ Samples. Phys. Solid State.

[45] Anishchenko I.V., Pokrovskii S.V., Rudnev I.A. (2018). Simulation of Magnetic Levitation Systems Based on Superconducting Rings. Bull. Lebedev Phys. Inst..

[46] Osipov M., Starikovskii A., Abin D., Rudnev I. (2019). Influence of the Critical Current on the Levitation Force of Stacks of Coated Conductor Superconducting Tapes. Supercond. Sci. Technol..

[47] Chair of Atomic Physics, Plasma Physics and Microelectronics. https://affp.phys.msu.ru/index.php/en/.

[48] Rudnev I., Abin D., Osipov M., Pokrovskiy S., Ermolaev Y., Mineev N. (2015). Magnetic Properties of the Stack of HTSC Tapes in a Wide Temperature Range. Phys. Procedia.

[49] Leon N. Cooper—Nobel Lecture. https://www.nobelprize.org/uploads/2018/06/cooper-lecture.pdf.

[50] Shnyrkov V.I., Yangcao W., Soroka A.A., Turutanov O.G., Lyakhno V.Y. (2018). Frequency-tunable microwave photon counter based on a superconducting quantum interferometer. Low Temp. Phys..

[51] Amosov A.P., Borovinskaya I.P. Powder Technology of Self-Propagating High-Temperature Synthesis of Materials. https://elibrary.ru/owgpcl.

[52] Prigozhin L., Sokolovsky V. (2011). Computing AC Losses in Stacks of High-Temperature Superconducting Tapes. Supercond. Sci. Technol..

[53] Rabadanova A.E., Gadzhimagomedov S.K., Palchaev D.K., Murlieva Z.K. (2022). Properties of YBCO Ceramics Depending on Oxygen Doping. Her. Dagestan State Univ..

[54] Pokrovskiy S., Mineev N., Sotnikova A., Ermolaev Y., Rudnev I. (2014). The Study of Relaxation Characteristics of Stack of HTS Tapes for Use in Levitation Systems and Trapped Flux Magnets. J. Phys. Conf. Ser..

[55] Abin D., Osipov M., Pokrovskii S., Rudnev I. (2016). Relaxation of Levitation Force of a Stack of HTS Tapes. IEEE Trans. Appl. Supercond..

[56] Bondarenko S.I., Krevsun A.V., Ilichev E.V., Hubner U., Koverya V.P., Link S.I. (2018). Thin Film Superconducting Quantum Interferometer with Ultralow Inductance. Low Temp. Phys..

[57] Kostyurina E.A., Kalashnikov K.V., Filippenko L.V., Kiselev O.S., Koshelets V.P. (2017). Highly symmetric superconducting quantum DC interferometer on Nb/AlO/Nb Josephson junctions for non-destructive testing systems of materials. Radio Eng. Electron..

[58] Korobova N.E., Ketegenov T.A., Tyumentseva O.A. (2009). Method for Producing High-Temperature Superconducting Films. Patent of the Republic of Kazakhstan.

[59] Tonoyan A.O., Arapkelyan E.R., Hayryapetyan S.M., Mamalis A.G., Davtyan S.P. (2001). On Some Issues of Obtaining Polymer-Ceramic Superconducting Kmpositions. Chem. J. Armen..

[60] Bologa M.K. (2006). Research and Innovations at the Institute of Applied Physics. Evolution and Achievements. Electron. Process. Mater..

[61] Schmidt V.V. (2013). The Physics of Superconductors: Introduction to Fundamentals and Applications.

[62] Sadovskii M.V. (2008). High-Temperature Superconductivity in Iron-Based Layered Compounds. Physics-Uspekhi.

[63] Popova E.N., Popov V.V., Romanov E.P., Sudareva S.V., Dergunova E.A., Vorobyova A.E., Balaev S.M., Shikov A.K. (2008). Effect of doping, composite geometry and diffusion annealing schedules on the structure of Nb_3_Sn layers in Nb/Cu-Sn wires. Defect Diffus. Forum.

[64] Levashov E.A., Rogachev A.S., Kurbatkina V.V. Promising Materials and Technologies of Self-Propagating High-Temperature Synthesis—Read in the Electronic Library System Znanium. https://znanium.ru/catalog/document?id=370115.

[65] Rosenband V., Gany A. (2014). Thermal Explosion Synthesis of a Magnesium Diboride Powder. Combust. Explos. Shock. Waves.

[66] Lebrat J.P., Varma A. (1991). Combustion Synthesis of the YBa_2_Cu_3_O_7−*x*_ Superconductor. Phys. C Supercond..

[67] Miloshenko V.E., Kalyadin O.V., Yu V. (2009). Izmailov Effect of magnetic field on freely moving superconductors in the audio frequency range. J. Tech. Phys..

[68] Pokrovskii S., Osipov M., Abin D., Rudnev I. (2016). Magnetization and Levitation Characteristics of HTS Tape Stacks in Crossed Magnetic Fields. IEEE Trans. Appl. Supercond..

[69] Romanovskii V.R. (2017). Thermal Mechanisms of Irreversible Destruction of Superconducting Properties of Technical Superconductors. J. Tech. Phys..

[70] Podlivaev A., Rudnev I. (2017). A New Method of Reconstructing Current Paths in HTS Tapes with Defects. Supercond. Sci. Technol..

[71] Romanovskii V.R. (2017). Thermoelectrodynamic Mechanisms of the Growth of Current–Voltage Characteristics of Technical Superconductors under Magnetic Flux Creep. Tech. Phys..

[72] Anischenko I.V., Pokrovskii S.V., Rudnev I.A. (2019). Simulation of Magnetization and Heating Processes in HTS Tapes Stacks. J. Phys. Conf. Ser..

[73] Anischenko I.V., Pokrovskii S.V., Rudnev I.A. (2019). The Dynamic Processes in Second Generation HTS Tapes under the Pulsed Current and Magnetic Impact. J. Phys. Conf. Ser..

[74] Kurbatova E., Kushchenko E., Kurbatov P. Comparison of Magnetic Systems with HTS Bulks and HTS Tape for Non-Contact Bearings. Proceedings of the 2020 21st International Symposium on Electrical Apparatus & Technologies (SIELA).

[75] Rusakov V.A., Melekh B.A.-T., Volkov M.P. (2020). Forming the Fe(Se1 –xTex) Superconducting Coatings on the Iron Surface. Tech. Phys..

[76] Antonov A.V., El’kina A.I., Vasiliev V.K., Galin M.A., Masterov D.V., Mikhaylov A.N., Morozov S.V., Pavlov S.A., Parafin A.E., Tetelbaum D.I. (2020). Experimental Observation of S-Component of Superconducting Pairing in Thin Disordered HTSC Films Based on YBCO. Phys. Solid State.

[77] About ISSP RAS. http://www.issp.ac.ru/main/index.php/en/about-issp.html.

[78] Kordyuk A.A. (2015). Pseudogap from ARPES Experiment: Three Gaps in Cuprates and Topological Superconductivity (Review Article). Low Temp. Phys..

[79] Kordyuk A.A., Krabbes G., Nemoshkalenko V.V., Viznichenko R.V. (2000). Surface influence on flux penetration into HTS bulks. Phys. B Condens. Matter.

[80] Borisenko S.V., Zabolotnyy V.B., Kordyuk A.A., Evtushinsky D.V., Kim T.K., Carleschi E., Doyle B.P., Fittipaldi R., Cuoco M., Vecchione A. (2012). Angle-resolved photoemission spectroscopy at ultra-low temperatures. J. Vis. Exp. JoVE.

[81] Solovyov I.I., Kornev V.K., Klenov N.V., Sharafiev A.V., Kalabukhov A.S., Chuharkin M.L., Snigirev O.V. Microwave Amplifier Based on a High-Temperature SQUID with Four Josephson Junctions—Patent|ISTINA—Intellectual System for Thematic Research of Scientometric Data. https://istina.ips.ac.ru/patents/5382370/.

[82] Degtyarenko P.N., Sadakov A.V., Ovcharov A.V., Degtyarenko A.Y., Gavrilkin S.Y., Sobolevskiy O.A., Tsvetkov A.Y., Massalimov B.I. (2023). Influence of the Gd Concentration on Superconducting Properties in Second-Generation High-Temperature Superconducting Wires. Pisʹma V Ž. Êksperimentalʹnoj Teor. Fiz..

[83] Kir’yakov N.V., Grigoryan E.A., Sikharulidze G.G., Morozov Y.G., Nersesyan M.D., Peresada A.G., Merzhanov A.G. Investigation into the Processes of Gas Evolution from HTSC-Ceramics Y-Ba-Cu-O During Vacuum Heat Treatment. https://www.researchgate.net/publication/305683658.

[84] Potanin A.Y., Levashov E.A., Kovalev D.Y. (2016). Dynamics of Phase Formation During Synthesis of Magnesium Diboride from Elements in Thermal Explosion Mode. Powder Metall. Funct. Coat..

[85] Opata Y.A., Monteiro J.F.H.L., Jurelo A.R., Siqueira E.C. (2018). Critical Current Density in (YBa_2_Cu_3_O_7−δ_)_1−*x*_–(PrBa_2_Cu_3_O_7−δ_)_x_ Melt-Textured Composites. Phys. C Supercond. Its Appl..

[86] Ahn B.T., Beyers R.B., Lee W.Y. Methods for Producing Tl2Ca2Ba2Cu3 Oxide Superconductors 1994. https://patents.google.com/patent/US5306698.

[87] Tolendiuly S., Fomenko S.M., Abdulkarimova R.G., Mansurov Z.A., Dannangoda G.C., Martirosyan K.S. (2016). The Effect of MWCNT Addition on Superconducting Properties of MgB2 Fabricated by High-Pressure Combustion Synthesis. Int. J. Self-Propagating High-Temp. Synth..

[88] Tolendiuly S., Fomenko S.M., Dannangoda G.C., Martirosyan K.S. (2017). Self-Propagating High Temperature Synthesis of MgB_2_ Superconductor in High-Pressure of Argon Condition. Eurasian Chem.-Technol. J..

[89] Tolendiuly S., Alipbayev K.A., Fomenko S.M., Sovet A., Zhauyt A. (2022). Effect graphite on magnesium diboride superconductivity synthesized by combustion method under argon pressure: Part I. Metalurgija.

[90] Tolendiuly S., Alipbayev K.A., Fomenko S.M., Sovet A., Zhauyt A. (2022). Effect graphite on magnesium diboride superconductivity synthesized by combustion method under argon pressure: Part II. Metalurgija.

[91] Tolendiuly S., Fomenko S.M., Abdulkarimova R.G., Akishev A. (2020). Synthesis and Superconducting Properties of the MgB_2_@BaO Composites. Inorg. Nano-Met. Chem..

[92] Tolendiuly S., Sovet A., Fomenko S. (2023). Effect of Doping on Phase Formation in YBCO Composites. J. Compos. Sci..

[93] Dautov L.M., Kalauov B.P., Kusainov S.K. (2006). New Tendencies in the Superconductivity Interpretation. I. Izv. Natsionalnoj Akad. Nauk Resp. Kazakhstan Seriya Fiz.-Mat..

[94] Afanasyev D.A., Ibraev N.K., Huangbai E., Kazakhstan Patent Database (2008). Method for Obtaining High-Temperature Superconducting Films. IP.

[95] Krasil’nikov V.N., Zhukov V.P., Chulkov E.V., Baklanova I.V., Kellerman D.G., Gyrdasova O.I., Dyachkova T.V. (2020). Novel method for the production of copper (II) formates, their thermal, spectral and magnetic properties. J. Alloys Compd..

[96] Dergunova E. Age of Superconductors. https://atomicexpert.com/age_of_superconductors.

[97] In Russia, a Mecca of Superconductors. https://www.iter.org/node/20687/russia-mecca-superconductors.

[98] Golovashkin A.I., Zherikhina L.N., Tskhovrebov A.M., Izmailov G.N. (2012). Supersensitive SQUID/Magnetostrictor Detecting System. Quantum Electron..

[99] Maksimov E.G. (2010). About Ginzburg—Landau, and a Bit about Others. Physics-Uspekhi.

[100] Mineev V.P. About Landau Institute. https://www.itp.ac.ru/en/about/.

[101] Bardeen J., Cooper L.N., Schrieffer J.R. (1957). Theory of Superconductivity. Phys. Rev..

[102] Feigelman M.V., Kamenev A., Larkin A.I., Skvortsov M.A. (2002). Weak charge quantization on a superconducting island. Phys. Rev. B.

[103] Carbotte J.P. (1990). Properties of Boson-Exchange Superconductors. Rev. Mod. Phys..

[104] Tinkham M. (1975). Introduction to Superconductivity.

[105] Zhetpisbaev K., Kumekov S., Mohd Suib N.R., Abd-Shukor R. (2017). Effect of complex magnetic oxides nanoparticle on (Bi_1.6_Pb_0.4_)Sr_2_Ca_2_Cu_3_O_10_ superconductor prepared by co-precipitation method. AIP Conf. Proc..

[106] Zhetpisbaev K., Kumekov S., Mohd Suib N.R., Abu Bakar I.P., Abd-Shukor R. (2019). Effect of Co_0.5_Zn_0.5_Fe_2_O_4_ Nanoparticle on AC Susceptibility and Electrical Properties of YBa_2_Cu_3_O_7-δ_ Superconductor. Int. J. Electrochem. Sci..

[107] Korobova N.E., Ketegenov T.A., Tyumentseva O.A. (2009). Method for Producing High-Temperature Superconducting Films. Patent of the Republic of Kazakhstan.

[108] Tonoyan A.O., Davtian S.P., Martirosian S.A., Mamalis A.G. (2001). High-temperature superconducting polymer–ceramic compositions. J. Mater. Process. Technol..

[109] Lakhno V.D. (2021). Pseudogap Isotope Effect as a Probe of Bipolaron Mechanism in High Temperature Superconductors. Materials.

[110] (2022). Technology Developed for NICA Became Breakthrough in Applied Field. https://www.jinr.ru/posts/technology-developed-for-nica-became-breakthrough-in-applied-field.

[111] Mitsen K.V., Ivanenko O.M. (2017). Superconducting Phase Diagrams of Cuprates and Pnictides as a Key to Understanding the HTSC Mechanism. Physics-Uspekhi.

[112] Aksenov V.L. (2009). Pulsed Nuclear Reactors in Neutron Physics. Physics-Uspekhi.

[113] Avdeev M.V., Aksenov V.L. (2010). Small-Angle Neutron Scattering in Structure Research of Magnetic Fluids. Physics-Uspekhi.

[114] Maksimov E.G., Dolgov O.V. (2007). A Note on the Possible Mechanisms of High-Temperature Superconductivity. Physics-Uspekhi.

[115] Vinnikov L.Y., Veshchunov I.S., Sidel’nikov M.S., Stolyarov V.S., Egorov S.V., Skryabina O.V., Jiao W., Cao G., Tamegai T. (2019). Direct Observation of Vortex and Meissner Domains in a Ferromagnetic Superconductor EuFe_2_(As_0.79_P_0.21_)_2_ Single Crystal. JETP Lett..

[116] Pustovit Y.V., Prokopenko O.V., Kurdyumov G.V., Kordyuk A.A. (2020). Anomalous Downshift of Electronic Bands in Fe(Se, Te) in Superconducting State. Metallofiz. I Noveishie Tekhnologii.

